# *Alobophora sandrae* n. gen. n. sp. (Digenea: Caballerotrematidae) infecting *Arapaima gigas sensu lato* (Osteoglossiformes: Arapaimidae) with a revision of *Caballerotrema*, key to Caballerotrematidae, and updated phylogeny[Fn FN1]

**DOI:** 10.1051/parasite/2024054

**Published:** 2024-09-23

**Authors:** Kamila Cajiao-Mora, John H. Brule, Micah B. Warren, Steven P. Ksepka, Haley R. Dutton, Stephen A. Bullard

**Affiliations:** 1 Aquatic Parasitology Laboratory and Southeastern Cooperative Fish Parasite and Disease Laboratory, School of Fisheries, Aquaculture, and Aquatic Sciences, College of Agriculture, Auburn University 559 Devall Dr. Auburn AL 36849 USA; 2 CIBAV Research Group, Veterinary Medicine School, Agrarian Sciences Department, Universidad de Antioquia Medellín 050034 Colombia; 3 Department of Zoology, School for Environmental Sciences and Development, North-West University Private Bag X6001 Potchefstroom 2520 South Africa

**Keywords:** Taxonomy, Fish parasites, Colombia, Amazon, Pirarucú, Biogeography

## Abstract

We propose and describe *Alobophora sandrae* Cajiao-Mora & Bullard n. gen., n. sp. (Digenea: Caballerotrematidae) for specimens we collected from arapaima, *Arapaima gigas sensu lato* (Osteoglossiformes: Arapaimidae) in the Amazon River near Leticia, Colombia. *Alobophora* differs from *Caballerotrema* Prudhoe, 1960 by lacking head collar projections and by having clustered corner spines and a narrow head collar (4–5× wider than pharynx), whereas *Caballerotrema* has head collar projections, lacks clustered corner spines, and has a broad head collar (7–8× wider than pharynx). We reassign *Caballerotrema annulatum* (Diesing, 1850) Ostrowski de Núñez & Sattmann, 2002 to the new genus, as *Alobophora annulata* (Diesing, 1850) Cajiao-Mora and Bullard n. comb., and provide a supplemental description of *Caballerotrema brasiliense* Prudhoe, 1960 based on specimens we collected from arapaima. We also examined the holotype and a paratype of *Caballerotrema piscicola* (Stunkard, 1960) Kostadinova & Gibson, 2001 and concluded that *C. piscicola* is a junior subjective synonym of *C. brasiliense*. Our *28S* phylogeny recovered *A. sandrae* sister to *A. annulata*, with that clade sister to a clade comprising *C. brasiliense* and an innominate species of *Caballerotrema*. Caballerotrematidae was recovered sister to Echinostomatidae. We also provide a dichotomous key to caballerotrematids based on head collar projections, corner spine arrangement, proportional pharynx and head collar breadth, testes shape and arrangement, body surface spine shape and distribution, vitellarium distribution, and abundance of prostatic cells.

## Abstract (fr)

Nous proposons et décrivons *Alobophora sandrae* Cajiao-Mora & Bullard n. gen., n. sp. (Digenea : Caballerotrematidae) pour les spécimens que nous avons collectés chez l’arapaïma, *Arapaima gigas sensu lato* (Osteoglossiformes : Arapaimidae) dans le fleuve Amazone près de Leticia (Colombie). *Alobophora* diffère de *Caballerotrema* Prudhoe, 1960 par l’absence de projections du collier céphalique et par la présence d’épines angulaires groupées et d’un collier céphalique étroit (4 à 5 fois plus large que le pharynx), tandis que *Caballerotrema* présente des projections du collier céphalique, n’a pas d’épines angulaires groupées et a un collier céphalique large (7 à 8 fois plus large que le pharynx). Nous réaffectons *Caballerotrema annulatum* (Diesing, 1850) Ostrowski de Núñez & Sattmann, 2002 au nouveau genre, sous le nom d’*Alobophora annulata* (Diesing, 1850) Cajiao-Mora et Bullard n. comb., et fournissons une description supplémentaire de *Caballerotrema brasiliense* Prudhoe, 1960 basée sur des spécimens que nous avons collectés sur des arapaïmas. Nous avons également examiné l’holotype et un paratype de *Caballerotrema piscicola* (Stunkard, 1960) Kostadinova & Gibson, 2001 et avons conclu que *C. piscicola* est un synonyme subjectif junior de *C. brasiliense*. Notre phylogénie 28S a trouvé *A. sandrae* groupe-frère d’*A. annulata*, avec ce clade frère d’un clade comprenant *C. brasiliense* et une espèce non nommée de *Caballerotrema*. Les Caballerotrematidae ont été trouvés comme groupe-frère des Echinostomatidae. Nous fournissons également une clé dichotomique des Caballerotrematidae basée sur les projections du collier de la tête, la disposition des épines d’angle, la largeur proportionnelle du pharynx et du collier de la tête, la forme et la disposition des testicules, la forme et la distribution des épines de la surface du corps, la distribution du vitellarium et l’abondance des cellules prostatiques.

## Introduction

Caballerotrematidae Tkach, Kudlai, and Kostadinova, 2016 (Digenea: Echinostomatoidea) [86] currently comprises four species infecting the intestine of freshwater fishes in the Amazon River Basin (ARB): *Caballerotrema brasiliense* Prudhoe, 1960 (type species) infecting arapaima (“paiché” in Peru; “pirarucú” in Brazil and Colombia), *Arapaima gigas* (Schinz) (Osteoglossiformes: Arapaimidae); *Caballerotrema aruanense* Thatcher, 1980 infecting arawana, *Osteoglossum bicirrhosum* (Cuvier) (Osteoglossiformes: Osteoglossidae); *Caballerotrema piscicola* (Stunkard, 1960) Kostadinova and Gibson, 2001 infecting arapaima and arawana; and *Caballerotrema annulatum* (Diesing, 1850) Ostrowski de Núñez and Sattmann, 2002 infecting electric “eels”, *Electrophorus* spp. (Gymnotiformes: Gymnotidae) [24, 43, 44, 66, 68, 83, 85]. Cajiao-Mora et al. [11] provided a list of known hosts and an inventory of all extant museum specimens representing Caballerotrematidae. Echinostomatoidea *sensu* Tkach et al. [86] comprises 8 families: Caballerotrematidae, Echinostomatidae Loss, 1899, Fasciolidae Railliet, 1895, Cyclocoelidae Stossich, 1903, Philophthalmidae Looss, 1899, Himasthlidae Odhner, 1910, Echinochasmidae Odhner, 1910, and Psilostomidae Looss, 1900 [53, 54, 64, 69, 82]. Two additional trematode families that infect marine turtles (Rhytidodidae Odhner, 1926; Calycodidae Dollfus, 1929 [26, 65]) are considered to belong to Echinostomatoidea [45]. Caballerotrematidae is the only family comprised solely of trematodes that mature in freshwater fishes (no caballerotrematid life cycle has been published to our knowledge), whereas the other families comprise species that predominantly mature in birds and mammals and less so reptiles and a few fishes [11, 86]. Only three non-caballerotrematid echinostomatoids are known to mature in fishes. *Himasthla elongata* (Mehlis, 1831) Dietz, 1909 (Himasthlidae) (as *H. tensa* [Linton, 1940]) infects the intestine of the Atlantic cod (a marine fish), *Gadus morhua* Linnaeus (Gadiformes: Gadidae) in the northwestern Atlantic Ocean off North America [9, 19, 25, 51]. *Singhia thapari* (Singh, 1953) Yamaguti, 1958 (Echinostomatidae) infects clown knifefish, *Chitala chitala* (Hamilton) (Osteoglossiformes: Notopteridae) from India, and *Singhia kruinensis* Lim and Furtado, 1985 (Echinostomatidae) infects both clown knifefish and bronze featherback, *Notopterus notopterus* (Pallas) (Notopteridae) in Malaysia [50, 80, 94]. Collectively, these records show a propensity for the fish-infecting echinostomatoids to mature in bonytongues (Osteoglossiformes): four nominal caballerotrematids infect two South American bonytongues and two echinostomatids infect two Asian bonytongues.

There are six accepted families of Osteoglossiformes: Osteoglossidae, Arapaimidae, Pantodontidae, Gymnarchidae, Mormyridae, and Notopteridae. Berra [6] provided a comprehensive summary of the biogeography of osteoglossiforms. Arapaimidae, the focus taxon for the present study, comprises two genera. The formerly monotypic *Arapaima* Müller and the African bonytongue *Heterotis niloticus* Cuvier. Bonytongues have a theorized Gondwanian distribution (all extant species distributing in the Southern Hemisphere) with fossil representatives dating to the Mesozoic (260–66 million yr ago) [6, 16, 39, 49]. However, fossils and nucleotide evidence suggest that vicariance alone does not explain osteoglossomorph distribution [48, 49].

The host we report herein, “*Arapaima gigas*” *sensu lato* (Arapaimidae), is among the largest freshwater fishes in the world, reaching 3 m in total length and 250 kg [73]. Arapaimas are piscivorous, facultative air-breathing fish with a swim bladder that functions as a breathing organ [8]. They naturally range in the central Amazon region: the floodplain of the rivers Araguaia-Tocantins, Solimões-Amazonas in Brazil, Colombia, Peru, and in the Essequibo and Rupununi river system of Guiana [13, 14, 38]. Their theorized invasive distribution (human-mediated) comprises rivers in Peru, Brazil, Bolivia, India, and Indonesia [15, 28, 47, 58, 61, 71]. These iconic fish are culturally and commercially important [36]. Their abundances are alleged to be declining as indicated by decreased landings at artisanal fish markets [36, 38]. Nevertheless, arapaima has been catalogued as “Data Deficient” since 1996 in the Red List of Threatened Species of the International Union for Conservation of Nature (IUCN) [93]. The Convention on International Trade in Endangered Species of Wild Fauna and Flora (CITES) [89] lists it as Appendix II (“Not necessarily now threatened with extinction but could become so unless trade is closely controlled”). Some authors have classified arapaima as a “conservation paradox” because of its “threatened” status in its native range (Brazil) and its invasive status in its theorized invasive range [15, 58].

The current taxonomic status of “*Arapaima gigas*” is contentious. Günther [34] synonymized the three species of *Arapaima* described by Valenciennes [90] i.e., *Arapaima agassizii* Valenciennes and *Arapaima mapae* Valenciennes from Brazil plus *Arapaima arapaima* Valenciennes from the Rupununi River in Guyana. Stewart [81] rejected the synonymies of Günther [34] and described a fifth species (*Arapaima leptosoma* Stewart) from the Solimões River, Amazonas, Brazil, based upon a combination of features associated with the morphology of the preopercle and dorsal fin. Although Fricke et al. [30] accepted the 5 species of *Arapaima*, the vast majority of modern, non-taxonomic literature pertaining to arapaimas still references the single species “*A. gigas*” in the broad sense (*sensu lato*). Genetic studies of arapaimas in the ARB indicated the presence of metapopulations, not distinct arapaima species [3, 29, 37, 63, 87, 91]. This evolving understanding of arapaima evolution and population biology makes the study of their parasites intriguing since parasites can be indicators of cryptic host species.

The metazoan parasites reported from arapaima comprise ~20 genera collectively representing species of Nematoda, Platyhelminthes (Digenea, Monogenoidea, Cestoidea) Acanthocephala, and Crustacea. The majority of those reports are from aquaculture ponds in Brazil and Peru [2, 4, 7, 23, 31, 57, 59, 60, 62, 75, 79, 84], and collections of parasites from wild-caught arapaimas are infrequent and few [46, 85]. Additional reports source from cultured or indeterminate populations of arapaimas [5, 68, 77, 78]. Some of those records are unaccompanied by a morphological diagnosis for the parasite species they report, a voucher specimen, a nucleotide sequence, or an image/illustration that supports the species identification.

Herein, we provide a description of a new species of Caballerotrematidae, propose a new genus for the new species, and propose a new synonymy for a closely related species. We also provide and updated phylogenetic analysis with the first sequences of the type species of *Caballerotrema*.

## Materials and methods

### Ethics

All applicable institutional, national, and international guidelines for the care and use of animals were followed. Fishes were acquired post-mortem from the local market.

### Parasite collection

Two fresh-dead, iced arapaima and the intestine of another fresh-dead, iced arapaima were collected opportunely from Plaza de Mercado (4°12′56.29″S 69°56′40.15″W), Leticia, Amazonas, Colombia in October 2023. Each intestine was dissected such that the intestinal mucosa was exposed before being placed in an acrylic settling column, exposed to tap water heated to 60 °C, and then rinsed by grasping the intestine with hemostats and repeatedly and rapidly dunking the intestine in the hot water. The intestine was then examined in a dish of clean citrated saline solution using a stereo-dissecting microscope with fiber optic lights and sub-stage illumination. Simultaneously, the washed contents of the intestine were allowed to settle in the acrylic column. After ~10 min, the flocculant material and fluid in the acrylic column was decanted and fresh water added. The sediment of the acrylic column then was pipetted incrementally into a clean glass petri dish with tap water and examined using a stereo-dissecting microscope. Heat-killed trematode specimens from the petri dish were then transferred with an artist brush or pipette into 10% neutral buffered formalin (n.b.f.) for morphology or into 95% non-denatured ethanol (EtOH) for DNA extraction. Fixed specimens were rinsed in water then stained overnight in Van Cleave’s hematoxylin with several drops of Ehrlich’s hematoxylin, dehydrated with a graded EtOH series, made basic at 70% EtOH with lithium carbonate and n-butylamine, dehydrated in absolute EtOH, cleared with clove oil, and permanently mounted on glass slides using Canada balsam and a coverslip. These specimens were drawn using an Olympus BX51 compound microscope (Olympus, Tokyo, Japan) equipped with differential interference contrast optical components and a drawing tube. Measurements were obtained using a Jenotipik Gryphax camera (Jenotipik AG, Jena, Germany) and are reported in micrometers (μm; unless otherwise stated) as the range followed by the mean, standard deviation, and sample size in parenthesis (Tables 1 and 2). Only spines in near optimal or optimal lateral or dorso-ventral view were measured (Table 2). Morphological terms and nomenclature for the genus follow Prudhoe [68], Ostrowski de Núñez and Sattmann [66], and Kostadinova and Gibson [44], except for the terminology of the esophagus that follows Truong et al. [88]. Terminology for the head collar follows Kanev et al. [40] and Cajiao-Mora et al. [11], and shape names follow Clopton [18]. Type specimens (1 holotype and 2 paratypes) of the new species and 4 vouchers of *C. brasiliense* were deposited in the National Museum of Natural History’s Invertebrate Zoology Collection (USNM, Smithsonian Institution, Washington, DC). An additional paratype of the new species and 2 vouchers of *C. brasiliense* were deposited in the Animal Parasitological Collection (APC, Agrarian Science Department, Universidad de Antioquia, Medellín, Antioquia, Colombia).

**Table 1:** Caballerotrematidae comparative measurements.

Species	*Caballerotrema brasiliense*	*C. brasiliense*	*C. brasiliense* (USNM1339898 as *Himasthla piscicola*)	*Alobophora annulata* n. comb.	*A. annulata*	*Alobophora sandrae* n. gen., n. sp.
Host	*Arapaima gigas*	*A. gigas*	*A. gigas*	*Electrophorus electricus*	*E.* cf. *varii*	*A. gigas*
	[44]	Present study	Present study	[66]	[11]	Present study
TBL (mm)	3.2–4.5	5.4–7.5 (6.7 ± 0.9; 4)	7.3–10.0 (8.7 ± 1.9; 2)	6.9–7.4 (7.1)	7.5–8.7 (8.1 ± 849; 2)	5.2–8.7 (7.6 ± 1.6; 4)
BMW	634–803	741–811 (773 ± 29; 4)	830–864 (847 ± 24; 2)	480–560 (520)	452–502 (474 ± 25; 3)	673–920 (785 ± 95; 5)
WEB	–	517–603 (575 ± 40; 4)	–	368–416 (392)	322–369 (346 ± 24; 3)	510–752 (637 ± 118; 5)
WO	–	423–627 (525 ± 85; 4)	502–724 (613 ± 157; 2)	–	418–459 (439 ± 21; 3)	407–548 (484 ± 69; 5)
WC	–	113–250 (205 ± 64; 4)	141–168 (154 ± 19; 2)	–	255–260 (258 ± 4; 2)	163–248 (202 ± 43; 4)
HCL	361–535	402–442 (424 ± 17; 4)	467–505 (486 ± 27; 2)	–	233–247 (240 ± 7; 3)	357–485 (429 ± 56; 5)
HCW	528–1042	741–811 (773 ± 29; 4)	830–864 (847 ± 24; 2)	320–432 (395)	345–358 (353 ± 7; 3)	673–920 (785 ± 95; 5)
OSL	111–169	146–166 (155 ± 9; 4)	145–183 (164 ± 27; 2)	75–119 (97)	120–122 (121 ± 1; 3)	203–230 (213 ± 11; 5)
OSMW	111–183	126–138 (132 ± 5; 4)	133–190 (161 ± 40; 2)	94–126 (107)	75–78 (76 ± 2; 3)	148–210 (185 ± 26; 5)
OSBW		73–88 (79 ± 8; 3)	89 (1)	–	–	60–107 (89 ± 17; 5)
NCA	–	206–229 (217 ± 13; 4)	–	–	125–135 (129 ± 5; 3)	244–327 (294 ± 35; 5)
ESL	222–310	482–581 (539 ± 47; 4)	–	251–377 (318)	341–411 (377 ± 35; 3)	639–885 (783 ± 115; 5)
ESW	–	29–31 (30 ± 1; 4)	–	–	20–21 (20 ± 1; 3)	45–65 (56 ± 8; 5)
PrEL	14–28	46–64 (51 ± 9; 4)	53 (1)	107–170 (132)	35–46 (41 ± 3; 3)	153–264 (207 ± 47; 5)
PHL	155–239	178–208 (194 ± 13; 4)	231–234 (232 ± 2; 2)	119–144 (129)	113–131 (124 ± 10; 3)	200–278 (227 ± 31; 5)
PHW	99–127	75–81 (78 ± 3; 4)	100–120 (110 ± 14; 2)	94–138 (121)	71–86 (80 ± 8; 3)	130–172 (148 ± 16; 5)
PsEL		249–320 (297 ± 33; 4)	–	–	–	286–437 (362 ± 64; 5)
EB	–	665–739 (701 ± 37; 4)	–	–	469–506 (484 ± 19; 3)	950–1147 (1051 ± 90; 5)
CSL	–	878–1192 (1034 ± 153; 4)	1356–1495 (1426 ± 98; 2)	860–1143 (948)	324–428 (363 ± 57; 3)	882–1041 (965 ± 59; 5)
CSW	–	232–276 (252 ± 19; 4)	337–401 (369 ± 45; 2)	176–251 (209)	144–230 (188 ± 43; 3)	112–164 (131 ± 21; 5)
VSL	239–380	311–348 (326 ± 16; 4)	360–416 (388 ± 40; 2)	163–327 (245)	262–300 (283 ± 19; 3)	287–463 (373 ± 74; 5)
VSW	200–352	269–295 (281 ± 11; 4)	416–441 (428 ± 18; 2)	232–301 (268)	240–257 (248 ± 9; 3)	310–463 (376 ± 68; 5)
OVL	78–84	166–194 (180 ± 16; 4)	186–235 (210 ± 35; 2)	201–207 (205)	164–172 (168 ± 4; 3)	137–229 (172 ± 40; 5)
OVW	100–211	126–170 (152 ± 19; 4)	201–226 (213 ± 18; 2)	226–239 (232)	162–187 (176 ± 13; 3)	131–169 (148 ± 18; 5)
OA (mm)	–	3.0–4.1 (3.6 ± 0.6; 4)	4.4–5.5 (5.0 ± 0.8; 2)	–	3.3–3.8 (3.5 ± 254; 3)	1.9–3.5 (2.9 ± 0.6; 5)
OP (mm)	–	2.1–3.3 (2.9 ± 0.5; 4)	2.8–4.2 (3.5 ± 1.0; 2)	–	4–4.8 (4.4 ± 530;2)	3.1–5.7 (4.5 ± 1; 4)
OÖL	–	73–109 (94 ± 15; 4)	–	–	61–74 (66 ± 7; 3)	36–106 (67 ± 26; 5)
OÖW	–	50–83 (70 ± 15; 4)	–	–	36–43 (40 ± 4; 3)	52–82 (65 ± 13; 5)
VDB	–	256–404 (332 ± 61; 4)	375–415 (395 ± 28; 2)	–	245–311 (282 ± 34; 3)	276–319 (298 ± 22; 3)
VDW	–	19–35 (26 ± 8; 4)	28–46 (37 ± 13; 2)	–	50–82 (63 ± 17; 3)	14–27 (23 ± 8; 3)
VL (mm)	–	3.3–5.4 (4.6 ± 0.9; 4)	4.0–6.3 (5.1 ± 1.6; 2)	–	6.1–6.2 (6.1 ± 72; 2)	4.0–7.4 (5.9 ± 1.4; 5)
VA (mm)	–	1.5–1.9 (1.7 ± 0.1; 4)	3.3–3.3 (3.3 ± 11; 2)	–	1.5–2.4 (1.9 ± 502; 3)	1.2–1.9 (1.6 ± 0.3; 4)
VP	–	157–225 (199 ± 31; 4)	282–542 (412 ± 184; 2)	–	134–195 (165 ± 43; 2)	133–144 (140 ± 5; 4)
UL (mm)	–	2.6–3.8 (3.2 ± 0.6; 4)	3.8–4.9 (4.3 ± 0.7; 2)	–	2.7–3.4 (3.1 ± 350; 3)	1.2–2.5 (1.9 ± 0.5; 5)
EL	–	77–87 (83 ± 5; 4)	103 (1)	72–85 (78)	82–98 (92 ± 9; 3)	80–98 (89 ± 8; 5)
EW	–	48–54 (50 ± 3; 4)	58 (1)	50–85 (61)	54–87 (67 ± 18; 3)	62–72 (68 ± 5; 5)
ATL	300–493	449–715 (561 ± 113; 4)	677–819 (748 ± 100; 2)	458–534 (484)	363–375 (370 ± 6; 3)	486–779 (609 ± 139; 5)
ATW	72–155	158–188 (174 ± 13; 4)	172–194 (183 ± 16; 2)	270–333 (301)	201–235 (215 ± 18; 3)	170–229 (199 ± 27; 5)
PTL	278–549	463–813 (602 ± 152; 4)	733–813 (773 ± 57; 2)	534–628 (569)	418–465 (441 ± 24; 3)	453–784 (581 ± 138; 5)
PTW	95–169	155–172 (164 ± 9; 4)	147–210 (178 ± 45; 2)	251–283 (267)	209–233 (222 ± 12; 3)	146–224 (188 ± 30; 5)
ITS	–	0	0	–	60–265 (141 ± 109; 3)	128–244 (179 ± 49; 5)
CP	–	270–393 (339 ± 51; 4)	263–350 (306 ± 62; 2)	–	208–286 (247 ± 55; 2)	66–124 (92 ± 25; 4)
FB	–	590–617 (602 ± 12; 4)	748–884 (816 ± 96; 2)	560–720 (640)	442–544 (480 ± 56; 3)	961–1242 (1105 ± 133; 5)
PTF (mm)	–	1.4–2.0 (1.8 ± 0.2; 4)	1.8–2.7 (2.2 ± 0.6; 2)	1.7–2.7 (2.2)	2.8–3.4 (3 ± 410; 2)	1.9–3.8 (3.0 ± 0.8; 4)
**Percentage**						
FB%	12–18	8–11 (9 ± 2; 4)	9–10 (9 ± 1; 2)	9	5–6	14–18 (15 ± 2; 4)
PTF%	24–29	26–31 (28 ± 2; 4)	25–27 (26 ± 2; 2)	23–39	37–39	36–44 (39 ± 4; 4)
U%	29–33	42–52 (48 ± 5; 4)	49–53 (51 ± 3; 2)	–	40–42	23–31 (26 ± 3; 4)
HC%	–	6–8 (6 ± 1; 4)	5–7 (6 ± 2; 2)	–	3	5–7 (6 ± 1; 4)
OS%	–	2–3 (2 ± 0; 4)	2 (2)	–	1–2	2–4 (3 ± 1; 4)
PH%	–	35–37 (36 ± 1; 4)	–	–	33–43	24–32 (29 ± 4; 5)
ES%	–	8–9 (8 ± 1; 4)		–	3–5	10–12 (11 ± 1; 4)
POS%	–	40–50 (44 ± 5; 4)	38–43 (40 ± 3; 2)	–	54–55	52–66 (60 ± 6; 4)
**Tegument**						
1st SL	–	12–17 (14 ± 2; 4)	–	12–19	7.8–8.7 (8 ± 0.6; 3)	14–19 (16 ± 3; 3)
1st SW	–	3–4 (3 ± 1; 4)	–	–	5.8–6.1 (6 ± 0.2; 3)	4.5–5 (5 ± 0; 3)
2nd SL	–	16–21 (19 ± 2; 4)	–	–	13–22 (18 ± 5; 3)	11–15 (13 ± 3; 2)
2nd SW	–	6 (6 ± 0; 4)	–	–	11–21 (16 ± 5; 3)	4–5 (5 ± 0; 3)
3rd SL	–	–	–	19–28	16–26 (20 ± 5; 3)	–
3rd SW	–	–	–	–	19–20 (19 ± 0; 3)	–
4th SL	–	–	–	–	13–21 (18 ± 4; 3)	–
4th SW	–	–	–	–	12–20 (16 ± 4; 3)	–

*Estimated from published drawing.

Abbreviations: anterior testis length (ATL); anterior testis width (ATW); maximum body width (BMW); end of ceca to posterior end of body length (CP); cirrus–sac length (CSL); cirrus–sac width (CSW); esophagus bifurcation to anterior end of body length (EB); egg length (EL); esophagus length as a percentage of TBL (ES%); esophagus length (ESL); esophagus maximum width (ESW); egg width (EW); forebody length (FB); forebody as a percentage of TBL (FB%); head collar as a percentage of TBL (HC%); head collar length (HCL); head collar width (HCW); inter-testicular space length (ITS); nerve commissure to anterior end of body length (NCA); ovary to anterior end of body length (OA); oötype length (OÖL); oötype width (OÖW); ovary to posterior end of body length (OP); oral sucker length as a percentage of TBL (OS%); oral sucker length (OSL); oral sucker maximum width (OSMW); oral sucker base width (OSBW); ovary length (OVL); ovary width (OVW); pharynx length as a percentage of esophagus length (PH%); pharynx length (PHL); pharynx width (PHW); post-pharynx esophagus length (PsEL); post-ovarian space length as a percentage of TBL (POS%); pre-pharyngeal esophagus length (PPE); pre-pharynx esophagus length (PrEL); post–testicular field length (PTF); post–testicular ﬁeld as a percentage of TBL (PTF%); posterior testis length (PTL); posterior testis width (PTW); total body length (TBL); uterine field as a percentage of TBL (U%); length of body occupied by uterus (UL); beginning of vitellarium to anterior end of body length (VA); transverse vitelline duct in breadth (VDB); transverse vitelline duct width (VDW); vitellarium length (VL); end of vitellarium to posterior end of body length (VP); ventral sucker length (VSL); ventral sucker width (VSW); body width at end of ceca (WC); body width at esophageal bifurcation (WEB); body width at ovary level (WO); Tegument abbreviations: tegumental spines length (SL); tegumental spines width (SW); inter-annulation space length (IAS); first segment of body (from anterior end of body to posterior end of ventral sucker) (1st); second segment of body (from posterior end of ventral sucker to posterior end of ovary) (2nd); third segment of body (from posterior end of ovary to posterior end of posterior testis) (3rd); fourth segment of body (from posterior end of posterior testis to end of annulations) (4th).

**Table 2:** Caballerotrematidae head collar spines comparative measurements.

Species	*Caballerotrema brasiliense*	*C. brasiliense*	*C. brasiliense* (USNM1339898 as *Himasthla piscicola*)	*Alobophora annulata* n. comb.	*Alobophora sandrae* n. gen., n. sp.
Host	*Arapaima gigas*	*A. gigas*	*A. gigas*	*Electrophorus* cf. *varii*	*A. gigas*
	[44]	Present study	Present study	[11]	Present study
**Corner spines**					
MO length	115–121	91–105 (98 ± 6; 6)	87–89 (88 ± 1; 3)	84–98 (91 ± 5; 3)	117–156 (132 ± 13; 9)
width	21–24	19–26 (23 ± 2; 8)	20–23 (22 ± 2; 4)	16–20 (17 ± 2; 3)	21–34 (27 ± 4; 10)
LO length	88–91	77–92 (83 ± 6; 8)	71–75 (73 ± 2; 4)	86–99 (95 ± 5; 3)	109–154 (129 ± 13; 9)
width	24	17–25 (19 ± 3; 8)	16–19 (17 ± 1; 4)	17–19 (18 ± 1; 3)	24–30 (27 ± 2; 9)
MA length	97–105	74–84 (78 ± 5; 3)	71–76 (74 ± 4; 2)	89–96 (93 ± 3; 3)	108–135 (121 ± 9; 10)
width	20	17–22 (19 ± 2; 7)	18–22 (20 ± 3; 2)	17–19 (18 ± 1; 3)	24–33 (27 ± 3; 10)
LA length	70–76	66–81 (73 ± 6; 8)	66–68 (67 ± 1; 3)	94–100 (98 ± 2; 3)	109–143 (123 ± 12; 10)
width	18–21	15–20 (18 ± 2; 8)	14–17 (16 ± 2; 4)	17–19 (18 ± 1; 3)	20–30 (26 ± 3; 10)
MO–LO distance	51*	33–43 (39 ± 4; 5)	37–45 (43 ± 4; 4)	0	0
**Lateral spines**					
Length	71–78	62–77 (70 ± 4; 18)	59–62 (61 ± 2; 2)	75–85 (82 ± 3; 3)	98–130 (110 ± 8; 27)
Width	20–29	16–21 (18 ± 1.2; 31)	11–17 (15 ± 1.5; 20)	13–15 (14 ± 1; 3)	18–28 (22 ± 2; 58)
**Dorsal spines**					
Length	62–75	–	–	–	–
Width	19–22	12–17 (16 ± 1.6; 11)	12–18 (15 ± 3; 6)	–	14–27 (21 ± 4; 13)

*Estimated from published drawing.

Abbreviations: medio-oral (MO); latero-oral (LO); medio-aboral (MA); latero-aboral (LA).

### DNA extraction

A total of 4 EtOH-preserved specimens were used for DNA extraction and sequencing. Extraction was made using a DNeasyTM Blood and Tissue kit (QIAGEN, Hilden, Germany) following the manufacturer’s protocol, except that the proteinase-K incubation period was extended overnight. Once extracted, DNA concentration was measured using a NanoDrop-One Microvolume Spectrophotometer (Thermo Fisher Scientific Waltham, MA, USA), diluted to 50 ng/μL, and stored at −20 °C. The partial *28S* and *ITS2* were amplified using primers outlined in Anderson et al., Cribb et al., Lockyer et al., all in Cajiao-Mora et al. [1, 11, 20, 52]. PCR reactions were performed following Truong et al. [88]. DNA amplification was verified with a 1% agarose gel stained with ethidium bromide. PCR products were purified using a QIAquick PCR Purification Kit (QIAGEN) according to the manufacturer’s protocol, except that the last elution step was performed with autoclaved nanopure H_2_O. DNA sequencing was performed by Genewiz (South Plainfield, NJ, USA). Sequence assembly and analysis of chromatograms were performed with Geneious prime version 2023.2.1. Nucleotide sequences of the new species were 1526 base pairs (bp) for the *28S* and 404 bp for the *ITS2*; those of *C. brasiliense* were 1520 bp for the *28S* and 483 bp for the *ITS2*. The obtained *ITS2* sequences were not used in the present study because there are insufficient available data in GenBank to make comparisons. All sequence data were deposited in GenBank.

### Phylogenetic analysis

The ingroup taxa comprised our newly-generated sequences plus sequences representing 5 of the 8 echinostomatoid families *sensu* Tkach et al. [86]. The outgroup taxa included a sequence from each of three other echinostomatoid families. Sequences were aligned with the multiple alignment tool using fast Fourier trans-form (MAFFT) [41] and trimmed to the length of the shortest sequence (1160 bp [*28S*]). JModelTest 2 version 2.1.10 was implemented to perform a statistical selection of the best-fit models of nucleotide substitution based on Bayesian Information Criteria [21]. Aligned sequences were reformatted (from .fasta to .nexus) using the web application ALTER [33] to run Bayesian Inference (BI) analysis. BI was performed in MrBayes version 3.2.7a [72] using substitution model averaging (nst-mixed) and a gamma distribution to model rate-heterogeneity. Defaults were used in all other parameters. Three independent runs with 4 Metropolis-coupled chains were run for 5,000,000 generations, sampling the posterior distribution every 1000 generations. Convergence was checked using Tracer v1.6.1 [70] and the “sump” command in MrBayes: all runs appeared to reach convergence after discarding the first 25% of generations as burn-in. A majority-rule consensus tree of the post-burn posterior distribution was generated with the “sumt” command in MrBayes. The inferred phylogenetic tree was visualized using FigTree v1.4.4 [70] and further edited for visualization purposes with Adobe Illustrator (Adobe Systems).

Note that the authors of the new genus, new species and new combinations are different from the authors of the article, following Article 50 of the International Code of Zoological Nomenclature (ICZN).

## Results

Echinostomatoidea Looss, 1902

Caballerotrematidae Tkach, Kudlai & Kostadinova, 2016

### *Alobophora* Cajiao-Mora & Bullard n. gen. (Figs. 1, 2)

urn:lsid:zoobank.org:act:B79986CC-2014-4410-9EDE-5D98DD4C2458

Type species: *Alobophora sandrae* Cajiao-Mora & Bullard n. sp.

Other accepted species: *Alobophora annulata* (Diesing, 1850) Cajiao-Mora and Bullard n. comb.

Etymology: *Alobophora* refers to the absence of head collar projections characteristic of the new genus.

**Figure 1:**
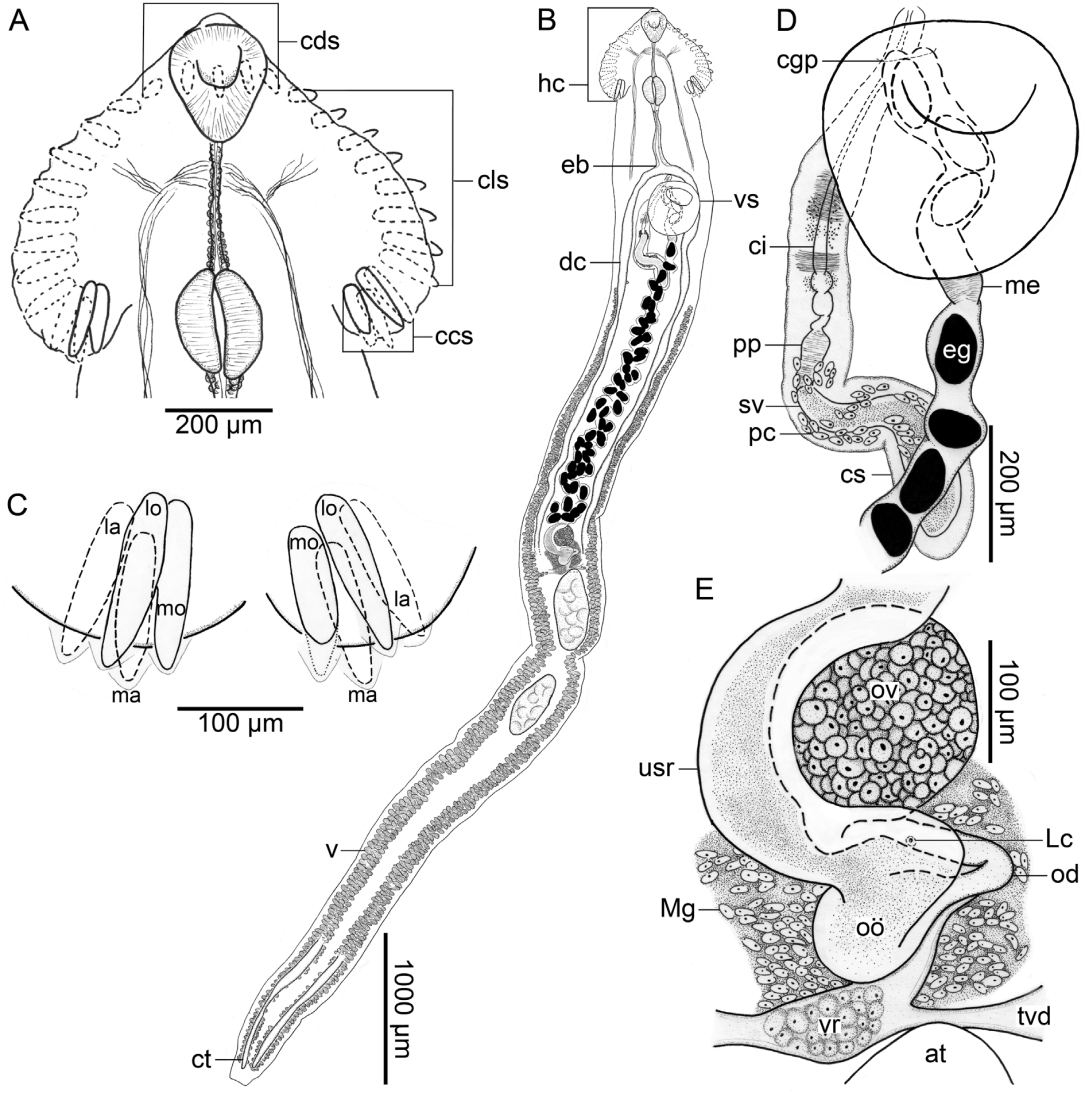
*Alobophora sandrae* Cajiao-Mora and Bullard n. gen., n. sp. (Digenea: Caballerotrematidae) (holotype USNM 1717984) from the intestine of an arapaima, *Arapaima gigas* (Schinz) *sensu lato* (Osteoglossiformes: Arapaimidae) from the Amazon River near Leticia, Amazonas, Colombia. Scale value beside bars. Ventral views. **A**, Head collar. **B**, Whole body. **C**, Ventral lobes of head collar. **D**, Male and female terminal genitalia. **E**, Detail of female genitalia. Abbreviations: anterior testis (at); cecum terminalia (ct); cirrus (ci); cirrus sac (cs); collar corner spines (ccs); collar dorsal spines (cds); collar lateral spines (cls); common genital pore (cgp); dextral cecum (dc); egg (eg); esophageal bifurcation (eb); head collar (hc); latero aboral (la); latero oral (lo); Laurer’s canal (Lc); medio aboral (ma); medio oral (mo); Mehlis’ gland (Mg); metaterm (me); oötype (oö); ovary (ov); oviduct (od); pars prostatica (pp); prostatic cells (pc); seminal vesicle (sv); transverse vitelline duct (tvd); uterine seminal receptacle (usr); ventral sucker (vs); vitellarium (v); vitelline reservoir (vr).

**Figure 2:**
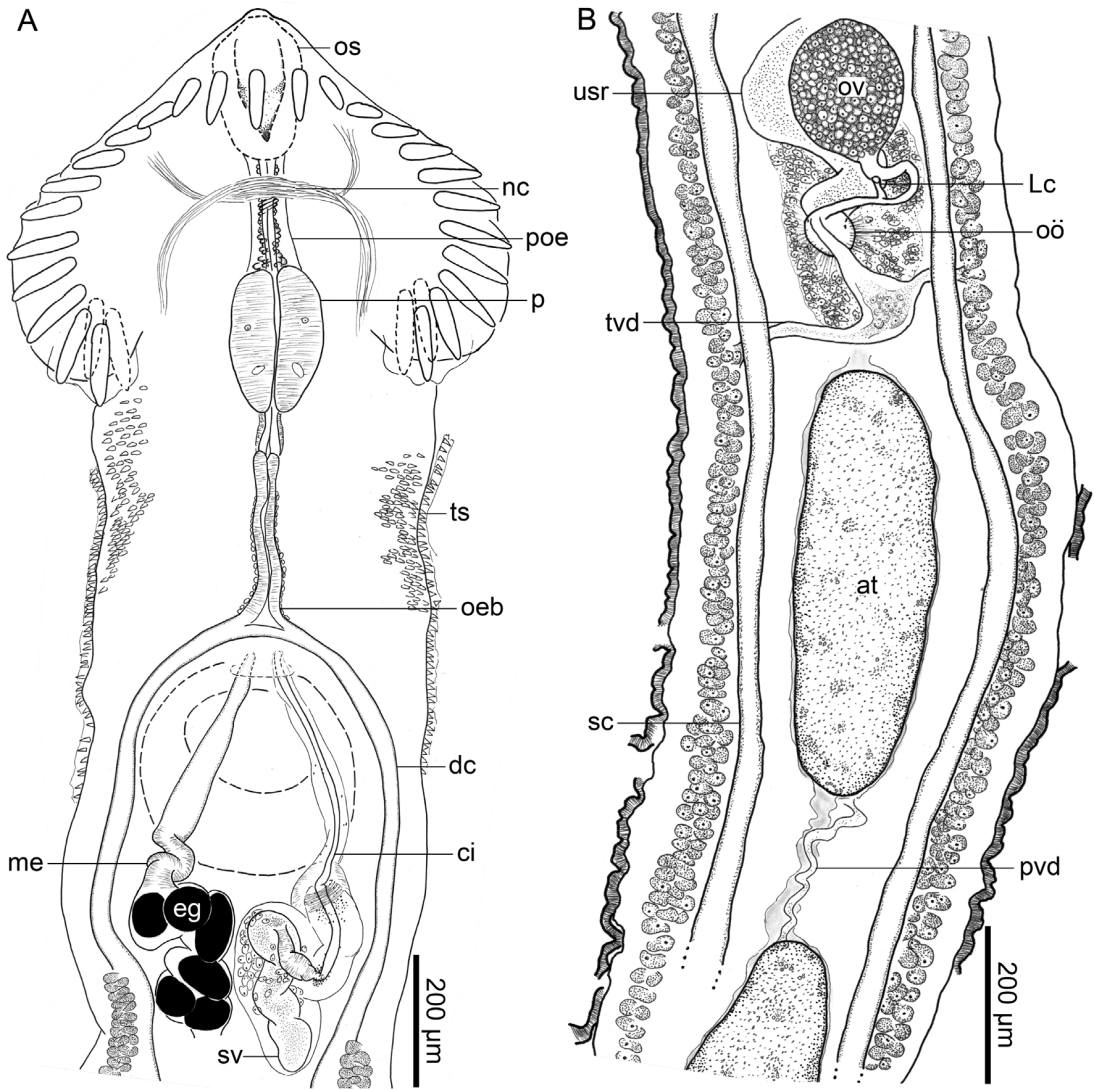
*Alobophora sandrae* Cajiao-Mora and Bullard n. gen., n. sp. (Digenea: Caballerotrematidae) (paratype 340-PA-FCA-UdeA) from the intestine of an arapaima, *Arapaima gigas* (Schinz) *sensu lato* (Osteoglossiformes: Arapaimidae) from the Amazon River near Leticia, Amazonas, Colombia. Scale value beside bars. Dorsal view. **A**, Anterior end of body. **B**, Third quarter of body. Abbreviations: anterior testis (at); body surface spines (bs); cirrus (ci); dextral cecum (dc); egg (eg); esophageal bifurcation (eb); Laurer’s canal (Lc); metaterm (me); nerve commissure (nc); oötype (oö); oral sucker (os); ovary (ov); pharynx (p); posterior vas efferent (pve); pre-pharyngeal esophagus (pe); seminal vesicle (sv); sinistral cecum (sc); transverse vitelline duct (tvd); uterine seminal receptacle (usr).

Diagnosis: Body elongate, dorsoventrally flat, widest at level of head collar (*A. sandrae*) or testes (*A. annulata*); forebody short. Tegument spinose; body surface spines scale-shaped, directing posteriad, decreasing (*A. sandrae*) or increasing (*A. annulata*) in size posteriad, restricted to anterior body half (*A. sandrae*; Fig. 2) or extending into posterior body half (*A. annulata*). Ventral sucker muscular, in first quarter of body. Head collar muscular, broader than long, wider (*A. sandrae*) or more narrow (*A. annulata*) than maximum body width, lacking head collar projections, spinose; head collar spines 29 in total, bullet-shaped, comprising dorsal spines, lateral spines, and corner spines; dorsal spines medial, 5 in number, distributing as a single dorsal row, middle spine at level of mouth, directing dorsally; lateral spines 8 in number per side of head collar (16 total), in a single row, directing laterad; corner spines comprising 2 pairs (4 corner spines) per side of head collar (8 total), directing posteriad; first pair comprising medio oral-aboral pair of spines; second pair comprising latero oral-aboral pair of spines, clustered with first pair of corner spines, directing posteriad. Oral sucker smaller than ventral sucker, ovoid. Pre-pharyngeal esophagus approximately same length (*A. sandrae*) or notably shorter (*A. annulata*) than pharynx; pharynx ovoid, at level of corner spines; pharynx: head collar ratio 1:5 (*A. sandrae*) or 1:4 (*A. annulata*); esophagus bifurcating dorsal or anterior to ventral sucker. Ceca 2 in number, simple, blind ending, extending posteriad in parallel with respective body margin, terminating in extreme end of body. Testes 2 in number, elongate (*A. sandrae*; Fig. 2B) or ovoid (*A. annulata*)*,* separate, in tandem, occupying third quarter of body. Cirrus sac large, dorsal to ventral sucker, extending posteriad to ventral sucker, without an enlarged middle portion (*A. sandrae*; Figs. 1D, 2A) or having an enlarged middle portion filled with prostatic cells (*A. annulata)*, containing seminal vesicle, pars prostatica, prostatic cells, and cirrus. Seminal vesicle sinuous or convoluted; pars prostatica tubular; prostatic cells low in number (*A. sandrae*; Figs. 1D, 2A), or comprising a dense mass (*A. annulata*) surrounding pars prostatica and anterior portion of seminal vesicle. Cirrus long, slender, aspinose. Genital pore ventral, in midline of body, posterior to esophageal bifurcation, dorsal to anterior ventral sucker margin. Genital atrium not observed. Ovary rounded, dextral, sinistral, or median, post-equatorial or equatorial. Oviduct emerging from posterior margin of ovary, slightly sinuous, dorsal to uterine seminal receptacle. Laurer’s canal short, emanating from proximal portion of oviduct, opening on dorsal surface of body, immediately posterior to ovary. Oötype ovoid, surrounded by compact Mehlis’ gland. Uterus comprising a short proximal portion and distal portion; proximal portion comprising uterine seminal receptacle, filled with sperm, ventral to oviduct and oötype, sinistral, dextral, or ventral to ovary; distal portion lacking sperm and having typical lumen, coiling anteriad between ceca and vitellarium; metaterm evident immediately anteriad to ventral sucker (*A. sandrae*; Figs. 1D, 2A) or indistinct (*A. annulata*). Vitellarium comprising 2 bilaterally symmetrical fields of vitelline follicles, enveloping ceca just in posterior half (*A. sandrae*; Fig. 1B) or in complete vitellarium length (*A. annulata*), distributing anteriad to cirrus-sac level (*A. sandrae*; Figs. 1B, 2A) or far posterior to cirrus sac (*A. annulata*), widening and becoming nearly confluent posteriorly; transverse vitelline duct posterior to oötype; vitelline reservoir connecting with oviduct via slender duct. Excretory pore terminal. Maturing in the intestine of primary division [6] South American freshwater fishes.

### *Alobophora sandrae* Cajiao-Mora & Bullard n. sp. (Figs. 1, 2)

urn:lsid:zoobank.org:act:AA42D552-D953-41C8-9385-5A4D5831EC96

Type host: Arapaima, *Arapaima gigas* (Schinz) *sensu lato* (Osteoglossiformes: Arapaimidae).

Site of infection: Intestine.

Type locality: Amazon River, Colombia.

Specimens deposited: Holotype USNM 1717984, paratypes USNM 1717985, 1717986, 340-PA-FCA-UdeA, 341-PA-FCA-UdeA.

Representative DNA sequences: *28S* GenBank accession No. PQ114582; *ITS2* GenBank accession No. PQ114583.

Etymology: The specific epithet “*sandrae*” honors Sandra Rocio Mora-Ramirez for her 27 years of service to the Instituto Amazónico de Investigaciones Científicas SINCHI.

Description (based on 4 whole-mounted mature specimens and the anterior end of a partial specimen. Measurements in Tables 1, 2): Body 8−11× (9 ± 1; 4) longer than wide, widest at level of head collar (Fig. 1B). Forebody representing 14%−28% (15% ± 2%; 4) of total body length. Body surface spines scale-shaped, having serrated posterior margin, decreasing in size and density posteriad, restricted to anterior body half. Tegument thick (Fig. 2A, 2B), containing body surface spines (Fig. 2A), decreasing in thickness posteriad (Table 2). Ventral sucker muscular, cup-shaped (Fig. 1D), in first quarter of body, 1−2× (2 ± 0; 5) longer than oral sucker, 2× (2 ± 0; 5) wider than oral sucker, anterior end overstanding anteriad and ventrally from body wall. Head collar deltoid in shape (Figs. 1A, 2A), constricted from body ventrally and laterally, spinose, lacking a ventral ridge, lacking head collar projections, 2× (2 ± 0; 5) wider than long, representing 5%−7% (6% ± 1%; 4) of total body length. Head collar spines 29 in total, comprising dorsal spines, lateral spines, and corner spines (Fig. 1A); dorsal spines 5 in number; lateral spines 8 in number per side of head collar (16 total); corner spines comprising 2 pairs (4 corner spines) per side of head collar (8 in total), radiating posteriad or posterolaterad from a common point where the first pair (medio oral-aboral) cluster with second pair (latero oral-aboral) (Fig. 1C). Nerve commissure dorsal to anterior pre-pharyngeal esophagus; nerve chords extending from nerve commissure anteriad and posteriad at sides of body (Figs. 1A, 1B, 2A). Oral sucker sub-terminal, ovoid, overstanding anteriad and ventrally from head collar, 2−3× (2 ± 1; 5) longer than wide, representing 2%−4% (3% ± 1%; 4) of total body length. Esophagus in midline of body, from posterior margin of oral sucker to almost reaching ventral sucker, comprising pre-pharyngeal glandular esophagus, pharynx, and post-pharyngeal muscular esophagus (Figs. 1A, 2A), representing 10%−12% (11% ± 1%; 4) of total body length. Pre-pharyngeal esophagus long, glandular (Fig. 2A), representing 24%−30% (26% ± 3%; 5) of total esophagus length. Pharynx ovoid, muscular, in middle portion of esophagus between corner spines (Figs. 1A, 1B, 2A), representing 24%−32% (29% ± 4%; 5) of total esophagus length, being 2−3× (2 ± 0.3; 5) wider than esophagus. Post-pharyngeal esophagus having two portions: anterior portion immediately post-pharyngeal, thin-walled, representing 7%−12% (9% ± 2%; 5) of total esophagus length (Fig. 2A); posterior portion having thick muscular wall, bifurcating into ceca, representing 35%−40% (37% ± 2%; 5) of total esophagus length; esophageal bifurcation anterior to ventral sucker (Figs. 1B, 2A). Ceca slender, extending posteriad, nearly reaching posterior end of body (Fig. 1B); posterior half of dextral and sinistral cecum enveloped by vitellarium (Fig. 1B).

Testes ovoid, horizontally elongated, in tandem, having smooth margins, in third quarter of body (Figs. 1B, 2B); each enveloped by a membrane (Fig. 2B); membrane delicate, thin, connecting testes, having short extensions anteriorly and posteriorly, resembling a mesentery tissue; posterior vas efferens dorsal to the membrane, evident in inter-testicular space; anterior vas efferens not observed; inter-testicular space representing 2%−3% (2% ± 0; 4) of total body length, post-testicular space representing 36%−44% (39% ± 4%; 4) of total body length. Cirrus sac elongated, sinuous (Fig. 1D), or convoluted (Fig. 2A), thin walled, from anterior margin of ventral sucker to beginning of vitellarium, mostly post-ventral sucker (Figs. 1D, 2A), containing seminal vesicle, pars prostatica, prostatic cells, and aspinose cirrus (Figs. 1D, 2A); seminal vesicle filled with seminal material, sinuous (Figs. 1D, 2A); pars prostatica slightly sinuous (Figs. 1D, 2A); prostatic cells low in number, not arranged in a compact mass, not enlarging the cirrus sac, surrounding distal seminal vesicle and pars prostatica (Figs. 1D, 2A); cirrus long, slender, dextral, not heavily muscular (Figs. 1D, 2A); genital pore ventral, in midline of body, posterior to esophageal bifurcation (Figs. 1D, 2A).

Ovary spheroid, having smooth borders, sinistral (Fig. 1E; *n* = 2), dextral (Fig. 2B; *n* = 2), or dorsal (*n* = 1) to uterine seminar receptacle, pre-testicular, in third quarter of body (Figs. 1B, 2B); pre-ovarian space representing 38%−43% (40% ± 2%; 4) of total body length; post-ovarian space representing 52%−66% (60% ± 6%; 4) of total body length. Oviduct emerging from posterior margin of ovary (Figs. 1D, 2B), tubular, sinuous. Laurer’s canal short, emanating from middle portion of oviduct, having dorsal opening (Figs. 1D, 2B). Oötype ovoid in shape, 2−4× (3 ± 1; 5) smaller than ovary, surrounded by compact Mehlis’ gland, ventrally connected to uterine seminal receptacle (Figs. 1D, 2B). Uterus coiling anteriad between ceca and vitellaria, ranging from oötype to almost reaching esophageal bifurcation, having numerous eggs (Fig. 1B), occupying 23%−31% (26% ± 3%; 4) total body length; proximal portion containing sperm, serving as uterine seminal receptacle (Figs. 1E, 2B); distal portion muscular, comprising a metaterm, sinistral, evident post ventral sucker, difficult to differentiate dorsal to ventral sucker (Figs. 1A, 2D). Vitellarium distributing in 2 bilaterally symmetrical fields of follicles, ranging from proximal cirrus sac to nearly reaching end of body, enveloping ceca in posterior body half, with each respective vitelline field becoming wider posteriad, occupying 73%−86% (78% ± 5%; 4) of body length (Fig. 1B); transverse vitelline ducts posterior to oötype, conjugating into vitelline reservoir, connecting with last portion of oviduct through a slender duct (Figs. 1E, 2B).

Excretory vesicle not observed, excretory pore terminal.

### *Caballerotrema* Prudhoe, 1960, emended

Type species: *Caballerotrema brasiliense* Prudhoe, 1960

Other accepted species: *Caballerotrema aruanense* Thatcher, 1980

**Figure 3:**
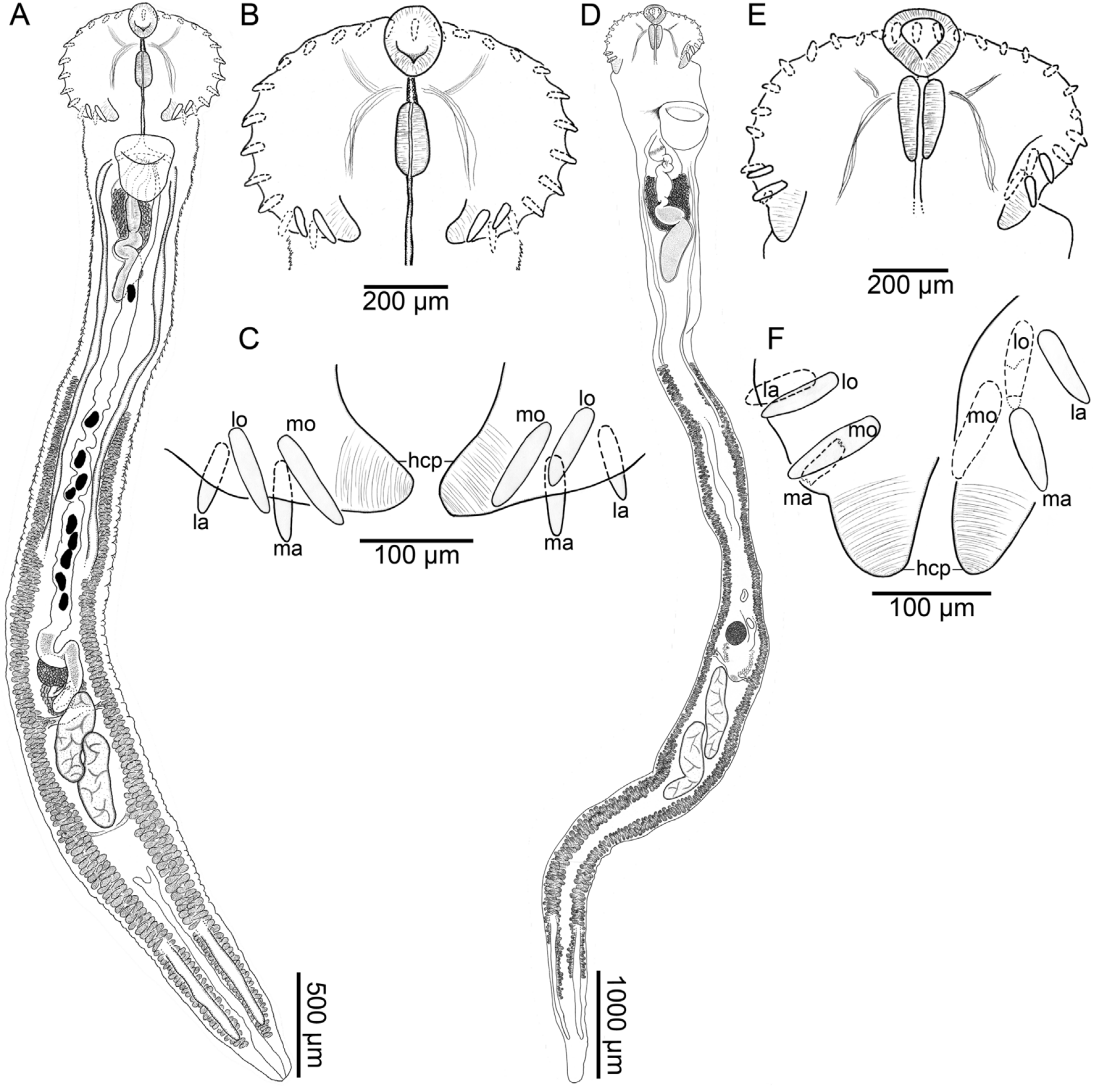
*Caballerotrema brasiliense* Prudhoe, 1960 (Digenea: Caballerotrematidae). Scale value beside bars. Ventral views. **A–C**, *C. brasiliense* (voucher USNM 1717987) from the intestine of an arapaima, *Arapaima gigas* (Schinz) *sensu lato* (Osteoglossiformes: Arapaimidae) from the Amazon River near Leticia, Amazonas, Colombia. **D–F**, *C. brasiliense* (as *C. piscicola*; type series USNM 1339898) from the intestine of an arapaima from an unknown locality in the Amazon River, Brazil. **A**, Whole body. **B**, Head collar. **C**, Ventral lobes of head collar. **D**, Whole body. **E**, Head collar. **F**, Ventral lobes of head collar. Abbreviations: head collar projections (hcp); latero aboral (la); latero oral (lo); medio aboral (ma); medio oral (mo).

Diagnosis: Body elongate, dorsoventrally flat in forebody, cylindrical in hindbody, widest at level of head collar; forebody short. Tegument spinose; body surface spines scale-shaped, directing posteriad, forming contiguous transverse rows, concentric (wrapping dorso-ventrally around body), in anterior body half (*C. brasiliense*) or body surface spines extension and morphology indeterminate (*C. aruanense*). Ventral sucker muscular, in first quarter of body. Head collar muscular, wider than long, wider than body, spinose; head collar projections present (Fig. 3C, 3F), not associated with a ventral ridge; head collar spines 29 in total, narrowly obdeltoid in shape, comprising dorsal spines, lateral spines, and corner spines; dorsal spines medial, 5 in number, distributing as a single row, middle spine at level of mouth, directing dorsally; lateral spines 8 in number per side of head collar, in a single dorsal row, directing laterad; corner spines directing posteriad, comprising 2 pairs (4 corner spines total) per side of body; first pair comprising the medio oral-aboral pair of spines; second pair comprising the latero oral-aboral pair of spines separated from first pair of corner spines (Fig. 3C, 3F). Oral sucker smaller than ventral sucker, ovoid. Pre-pharyngeal portion of esophagus short; pharynx ovoid, located far anterior to corner spines (Fig. 3B, 3E); esophagus bifurcating dorsal to ventral sucker. Ceca 2 in number, simple, blind-ending, extending posteriad in parallel with respective body margin, terminating in extreme posterior end of body. Testes 2 in number, elongate and sinuous (*C. brasiliense*; Fig. 3A, 3D) or oval (*C. aruanense*), contiguous and oblique (*C. brasiliense*) or separate and tandem (*C. aruanense*), occupying third quarter of body. Cirrus sac large, dorsal to ventral sucker, extending posteriad to ventral sucker, having enlarged middle portion filled with prostatic cells, containing seminal vesicle, pars prostatica, prostatic cells, and cirrus. Seminal vesicle straight or convoluted; pars prostatica tubular, lined with small anuclear blebs; prostatic cells comprising a dense mass surrounding pars prostatica, occupying middle portion of cirrus. Ejaculatory duct short. Cirrus short, aspinose. Genital pore ventral, medial, at level of esophageal bifurcation. Genital atrium not observed. Ovary rounded or transversally ovoid, dextral or median, post-equatorial or equatorial. Oviduct emerging from posterior margin of ovary, slightly sinuous, dorsal to uterine seminal receptacle. Laurer’s canal short, emanating from proximal portion of oviduct, opening on dorsal surface in dextral half of body immediately posterior to ovary. Oötype ovoid, surrounded by compact Mehlis’ gland, ventrally connected to uterine seminal receptacle. Uterus comprising a proximal portion and distal portion; proximal portion comprising uterine seminal receptacle, ventral to oviduct and oötype, sinistral to ovary; distal portion lacking sperm and having a typical lumen, coiling anteriad between ceca and vitellarium; metraterm indistinct. Vitellarium comprising 2 bilaterally symmetrical fields of vitelline follicles, enveloping ceca, distributing from mid-way between ventral sucker and ovary to posterior body end, widening and becoming nearly confluent posteriorly; transverse vitelline ducts posterior to oötype; vitelline reservoir connecting with oviduct via slender duct. Excretory pore terminal. Maturing in the intestine of primary division [6] South American freshwater fishes.

### *Caballerotrema brasiliense* Prudhoe, 1960 (Fig. 3)

Synonyms: *Caballerotrema piscicola* (Stunkard, 1960) Kostadinova and Gibson, 2002; *Himasthla piscicola* (Stunkard, 1960)

Type and only known host: arapaima, *Arapaima gigas* (Schinz) (Osteoglossiformes: Arapaimidae).

Site of infection: Intestine.

Type locality: Amazon River, Brazil.

Additional localities: Amazon River, Colombia.

Voucher material: USNM 1717987, 1717988, 1717989, 1717990; 342-PA-FCA-UdeA, 343-PA-FCA-UdeA, 344-PA-FCA-UdeA.

Representative DNA sequences: *28S* GenBank accession No. PQ114584, PQ114585; *ITS2* GenBank accession No. PQ114586.

Description (based on type material USNM 1339898; voucher specimens USNM 1373160, USNM 1373159; and newly collected specimens. Measurements in Tables 1, 2): Body 7−12× (9 ± 1; 6) longer than wide, widest at level of head collar (Fig. 3A, 3D). Forebody representing 8%−11% (9% ± 2%; 6) of total body length. Body surface spines scale-like shaped, having serrated posterior margin, increasing in size and decreasing in density posteriad, extending from head collar to middle section of body (Fig. 3A) (body surface spines absent in USNM 1339898). Ventral sucker muscular, funnel-shaped or cup-shaped, in first section of body (Fig. 3A, 3D). Head collar oval, 2× (2 ± 0; 6) wider than long, constricted from body ventrally and laterally, having head collar projections, having 29 bullet-shaped spines (Fig. 3B, 3E); head collar projections muscular, 2 in number, terminal, in posterior margin of head collar (Fig. 3C, 3F). Head collar spines 29 in number, comprising dorsal spines, lateral spines, and corner spines (Fig. 3B, 3E); dorsal spines 5 in number, dorsal to oral sucker; lateral spines 8 in number per side of head collar (16 in total), slightly smaller than corner spines; corner spines 4 in number per side of head collar (8 in total), anterior to head collar projections, arranged in two pairs: medio oral-aboral and latero oral-aboral (Fig. 3C, 3F), being the oral spines ventral to the head collar and the aboral spines dorsal to the head collar, being the medio oral spine the biggest one (Table 2); medio oral-aboral spines distanced from latero oral-aboral spines (Fig. 3C, 3F; Table 2).

Nerve commissure dorsal to pre-pharyngeal portion of esophagus (*n* = 3), or dorsal to pharynx (*n* = 1); nerve chords extending from nerve commissure anteriad and posteriad at sides of body (Fig. 3A, 3B, 3D, 3E).

Oral sucker sub-terminal, oval, overstanding from head collar (Fig. 3A, 3B, 3D, 3E). Pre-pharyngeal portion of esophagus short, representing 8%−13% (10% ± 2%; 4) of esophagus length (Fig. 3B, 3E). Pharynx in anterior portion of esophagus and middle portion of head collar (Fig. 3B, 3E), representing 35%−37% (36% ± 1%; 4) of esophagus length, 2−3× (3 ± 0.1; 4) wider than esophagus, pharynx: head collar width ratio 1:7–8. Esophagus having a thin muscular wall, median, representing 8%−9% (8% ± 1%; 4) of total body length, bifurcating into ceca; esophageal bifurcation dorsal to ventral sucker (Fig. 3A) (esophageal bifurcation not observed in USNM 1339898 [Fig. 3D]). Ceca slender, extending posteriad nearly reaching posterior end of body (Fig. 3A, 3D); dextral and sinistral cecum enveloped by vitellarium in posterior half of body (Fig. 3A, 3D).

Testes elongated, sinuous, contiguous, in third quarter of body (Fig. 3A, 3D); anterior testis overlapping with vitelline reservoir (Fig. 3A); posterior testis sinistral to anterior testis (Fig. 3A) or dextral to anterior testis (Fig. 3D); vasa efferentia not observed. Post-testicular space 25%−31% (27% ± 2%; 6) of total body length. Cirrus sac dorsal to ventral sucker, extending posteriad to ventral sucker (Fig. 3A, 3D), containing seminal vesicle, pars prostatica, aspinose cirrus, and prostatic cells (Fig. 3A, 3D), middle portion enlarged by prostatic cells (Fig. 3A, 3D); seminal vesicle tubular, sinuous, proximally surrounded by prostatic cells (Fig. 3A, 3D); pars prostatica tubular, straight, surrounded by prostatic cells; cirrus muscular; genital pore ventral, in midline of body, posterior to esophagus bifurcation (genital pore not observed in USNM 1339898 [Fig. 3D]).

Ovary spheroid, dextral to and overlapping with uterine seminal receptacle, in third quarter of body (Fig. 3A, 3D); pre-ovarian space 47%−61% (55% ± 4%; 6) of total body length; post-ovarian space 38%−50% (43% ± 4%; 6) of total body length. Oviduct emerging from posterior margin of ovary, slightly sinuous. Laurer’s canal emanating from middle portion of oviduct, having a dorsal opening, immediately post-ovarian. Oötype ovoid in shape, surrounded by compacted Mehlis’ gland, ventrally connected to uterine seminal receptacle (oviduct, Laurer’s canal, and oötype not observed in USNM 1339898). Uterus slightly sinuous, extending anteriad between ceca and vitellaria, from oötype to ceca bifurcation, having small number of eggs, occupying 42%−53% (49% ± 54%; 6) of total body length; proximal portion of uterus containing sperm, comprising uterine seminal receptacle (Fig. 3A); metaterm not observed. Vitellarium distributing in 2 bilaterally symmetrical fields of follicles, enveloping posterior half of ceca, extending from half-way between cirrus-sac and ovary to posterior end of body, occupying 54%−73% (66% ± 7%; 6) of body length (Fig. 3A, 3D); transverse vitelline ducts posterior to oötype, conjugating into vitelline reservoir, connecting with last portion of oviduct close to oötype.

Excretory vesicle Y-shaped (Fig. 3A), in last quarter of body; excretory pore terminal.

### Remarks

*Alobophora* differs from *Caballerotrema* by having a narrow head collar (4–5× wider than pharynx) and clustered corner spines and by lacking head collar projections, whereas *Caballerotrema* has a broad head collar (7–8× wider than pharynx), lacks clustered corner spines, and has head collar projections. We herein reassign *C. annulatum* to the new genus as *Alobophora annulata* (Diesing, 1850) Cajiao-Mora and Bullard n. comb. because it lacks head collar projections and because of the arrangement of its corner spines. Hence, we accept 2 species of *Alobophora*: the type species (*A. sandrae*) and *A. annulata*. The new species differs from its congener (*A. annulata*) by the combination of having a head collar that is wider than the maximum body width, body surface spines that decrease in size posteriad and terminate in the anterior body half (anterior to testes), a cirrus sac having few prostatic cells, and a vitellarium that extends anteriad to the cirrus sac. *Alobophora annulata* has a body that is wider than the head collar (body widest at level of testes), body surface spines that increase in size posteriad and terminate in the posterior body half (posterior to testes), a cirrus sac having many prostatic cells, and a vitellarium that is far posterior to the cirrus sac.

Although the new species is the fourth caballerotrematid described from arapaima, the previous 3 caballerotrematids have taxonomic problems. We accept *A. sandrae* and *C. brasiliense* but we regard *C. piscicola* as a junior subjective synonym of *C. brasiliense* and follow Kostadinova and Gibson [44] in considering *C. arapaimense* as a *species inquirendum*. The justifications for these nomenclatural actions are as follows. First, we consider *C. piscicola* a junior subjective synonym of *C. brasiliense* because it has head collar projections, a wide head collar (wider than maximum body width; 7× wider than pharynx), corner spines arranged as two separated pairs (Fig. 3F), sinuous and overlapping testes (Fig. 3D), and a vitellarium extending anteriad midway between the cirrus sac and the ovary (Fig. 3D). Prudhoe [68] described *C. brasiliense* infecting an arapaima from an unspecified location in the Brazilian ARB. That same year, Stunkard [83] described *C. piscicola* (as *H. piscicola*) from an arapaima also from an unspecified location in the Brazilian ARB. Stunkard’s [83] specimens were placed in water for some days before they were processed for whole-mounting. As Stunkard [83] indicated, we agree that these specimens likely partly deteriorated in water because the body shape is greatly extended and the body surface spines are missing (perhaps the spines detached as the specimens deteriorated in water) [83; p. 546]. He described the head collar as reniform-shaped but did not describe projections nor do the drawings show that feature (Fig. 15 in Stunkard [83]). Kostadinova and Gibson [44] reassigned *H. piscicola* to *Caballerotrema* but did not provide a morphological description of that species. We clearly discerned head collar projections in Stunkard’s [83] types (USNM 1339898) of *H. piscicola*, (Fig. 3D–3F); however, no additional detail of the female genitalia, esophagus, esophageal bifurcation, genital pore, and body surface could be discerned from these specimens (Fig. 3D). Second, the description of *C. brasiliense* by Prudhoe [68] was made with poorly fixed (contracted) specimens. We herein collected new specimens, fixed them properly, and were able to provide a detailed description of the head collar, head collar spines, genitalia (cirrus sac, seminal vesicle, oviduct, Laurer’s canal, oötype, vitellarium, and transverse vitelline duct), and body surface spines (distribution, measurements, and shape). This is the first diagnosed *C. brasiliense* tethered to a nucleotide sequence deposited in GenBank with voucher specimens in a lending museum. Third, regarding *C. arapaimense*, Thatcher [85] described this species from an arapaima from the Lago Janauacá, Manaus, Amazonas, Brazil. He differentiated the species from its congeners by having larger head collar corner spines. Based on the published description of *C. arapaimense*, Kostadinova and Gibson [44] stated that *C. arapaimense* could be a synonym of *C. brasiliense* because “*it agrees well with the description of the latter*” [44; p. 198]. Further, Kostadinova and Gibson [44] identified one of the paratypes of *C. arapaimense* (INPA 038 g) as *C. brasiliense* based on the size and shape of its collar spines. They also mentioned that the additional paratypes (INPA 038 e; INPA 038 h) probably belong to different *Caballerotrema* spp. based on the different sizes and shapes of their head collar spines [44; p. 198]. They considered *C. arapaimense* as a *species inquirendum* [44; p. 203], and we follow that herein.

### Phylogenetic results

**Figure 4:**
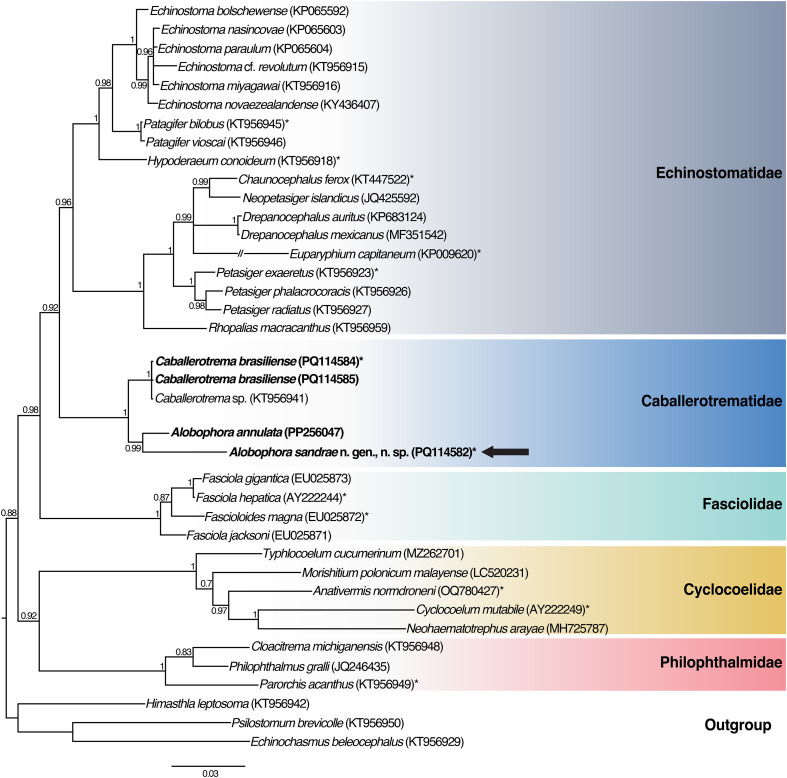
Large subunit ribosomal (*28S*) DNA phylogeny (Bayesian inference). Values beside nodes are posterior probability. Scale bar is in substitutions per site. GenBank accession numbers are in parenthesis following each taxon. Newly generated sequences of Caballerotrematidae (Digenea: Echinostomatoidea) are highlighted in bold. The new described genus and species is indicated by an arrow. Type species are indicated by asterisk (*).

Our amplified *28S* sequence representing *A*. *sandrae* comprised 1529 nucleotides (GenBank accession No. PQ114582) and was 97.1% similar (44 bp different) to that of *A. annulata* (GenBank accession No. PP256047; from *Electrophorus* cf. *varii* from the ARB in Leticia, Amazonas, Colombia). Our *28S* sequences of *C. brasiliense* (GenBank accession No. PQ114584; PQ114585) comprised 1520 nucleotides and are identical to each other. They were 99.8% similar (3 bp different) to that of *Caballerotrema* sp. (GenBank Accession No. KT956941; from *A. gigas* from the Peruvian Amazon). The BI analysis (Fig. 4) recovered our sequence of *A. sandrae* sister to that of *A. annulata* (GenBank accession No. PP256047). They both were recovered sister to a clade comprising our sequences of *C. brasiliense* and that of *Caballerotrema* sp. (GenBank accession No. KT956941). Both clades comprise Caballerotrematidae, which was recovered sister to Echinostomatidae (see Fig. 4). Our tree topology (Fig. 4) resembles that recovered by Tkach et al. [86]. It differs by having a better support value for the clade of Caballerotrematidae and Echinostomatidae (BI support values 0.99 vs 0.72). Both were recovered sister to Fasciolidae. Our tree topology differs from that of Cajiao-Mora et al. [11], who recovered Caballerotrematidae sister to Fasciolidae and both sister to Echinostomatidae, however, with low support (0.77).

### Key for Caballerotrematidae genera and species

**1a.** Head collar projections present; corner spines in two separated pairs; head collar 7–8× wider than pharynx …………………………………………………………………………………………………….. ***Caballerotrema* (2)**

**1b.** Head collar projections absent; corner spines in two clustered pairs; head collar 4–5× wider than pharynx ……………………………………………………………………………………………...………... ***Alobophora* (3)**

**2a.** Testes sinuous and abutting or overlapping …………………………………………..………… ***C. brasiliense***

**2b.** Testes oval and separated …………………………………………………………….………… ***C. aruanense***

**3a.** Head collar wider than maximum body width; body surface spines decreasing in size posteriad and terminating in anterior body half (anterior to testes); vitellarium extending anteriad reaching level of cirrus sac; having few prostatic cells .………………………………………………..……….……………………..………. ***A. sandrae***

**3b.** Head collar more narrow than maximum body width; body surface spines increasing in size posteriad and terminating in posterior body half (posterior to testes); vitellarium far posterior to cirrus sac; having many prostatic cells …………………………………………………………………………………………….………… ***A. annulata***

## Discussion

The biogeography of osteoglossiform fishes makes the study of their parasites interesting. Some authors have proposed that particular parasites of osteoglossiforms were “Gondwana relicts” [55, 56], e.g., the cestode genus *Nesolecithus* Poche, 1922 (Amphilinidea) [32, 67] and the nematode genus *Nilonema* Khalil, 1960 (Philometridae) [42, 76]. However, recent studies have challenged the hypothesis of the breakup of Gondwana as the origin of the distribution pattern in Osteoglossiformes and several other classically called “Gondwanaland” taxa [22, 49, 74]. A fossil-based estimate of origin time for Osteoglossomorpha ranges from the Late Triassic to the Middle Jurassic [12]. The clade is old enough to have been affected by the breakup of Gondwana, and even Pangea. Nevertheless, Capobianco and Friedman [12] stressed that osteoglossomorphs are characterized by a complex biogeographic history that involved several long-distance dispersals as well as continental vicariance and that has been partially hidden by regional extinctions [12; p. 683]. These intriguing aspects of osteoglossiform natural history could be explored independently by studying their parasites if the biodiversity of their parasites was better known. For example, analogous studies have used parasite taxonomic and phylogenetic evidence to test the marine incursion hypothesis of turtles in South America [10]; others explore patterns and processes of historical biogeography by combining phylogenetics and biogeography of fishes (sturgeons) and their parasites with the geological history of the Earth [17].

The identification of the arapaimas we dissected was indeterminate and nuanced based on recent taxonomic and genetic work with arapaimas. Studies on population genetics of arapaima conducted in the main stem of the Amazon River (Peru, Colombia, Brazil) and the Araguaia-Tocantins River basin (Brazil) concluded that genetic data do not support the five described species [3, 29, 37, 63, 87, 91]. Instead, they suggested that arapaimas show structured populations with low gene flow, a high level of relatedness, inbreeding, and reduced genetic variability [3, 15, 29, 37, 63, 87, 91]. The results have been linked with the potential occurrence of population bottlenecks associated with genetic drift, historical reductions in stocks, the sedentary behavior of the species, and the characteristics of each basin and its floodplain dynamics [3, 63, 91]. The pattern of genetic structure of arapaima has also been associated with the evolution of the landscape of the Amazon region [63]. This is supported for arapaima populations inhabiting the lowland intercatonic basin and the Brazilian shield of the ARB. However, a study conducted in southwestern Guyana (Essequibo and Branco River basins in the Guyana shield) indicated allopatric differentiation, suggesting sympatric species inhabiting the Essequibo and Pirara Rivers [92]. Hence, while the parasites of these fishes are important and could advance our understanding of arapaima natural history, we strongly suggest that a genetic voucher, photographs, and meristics are curated along with the parasite voucher or type specimens.

Infections of *Caballerotrema* spp. are known from cultured arapaima [4, 23, 79, 84]. Delgado et al. [23] reported *C. arapaimense* from the stomach of juvenile and adult arapaimas in Peru. Those records are unaccompanied by a voucher specimen or a morphological diagnosis for the parasite reported. Further, *C. arapaimense* has been considered a *species inquirendum* since Kostadinova and Gibson [44]. Hence, the identification of *C. arapaimense* infecting cultured arapaimas in Peru remains dubious. Serrano-Martínez et al. [79] and Tafur and Cotrina [84] reported *C. brasiliense* and *Caballerotrema* sp., respectively, infecting the intestine of juvenile arapaimas cultured in the Peruvian Amazon. Fewer reports exist in wild arapaimas. Dos Santos et al. [27] identified *C. brasiliense* infecting the intestine of arapaimas from the Araguaia River, Mato Grosso, Brazil. Voucher specimens are curated at the Instituto Oswaldo Cruz Helminthological Collection [27]. Additional records include Prudhoe [68], while erecting *Caballerotrema* from an arapaima from an unknown locality in Brazil, and Thatcher [85], who described *C. brasiliense* and *C. arapaimense* from arapaimas from Lago Janauacá, Manaus, Amazonas, Brazil. Comprehensive and detailed descriptions, nucleotide sequences, and voucher material curated in a museum are important for taxonomy. Prior to this study, no comprehensive morphological and nucleotide analysis had been conducted on arapaima caballerotrematids. The present description of *A. sandrae* and the identification of *C. brasiliense* represent the first records of caballerotrematids infecting arapaimas in the Colombian Amazon River.

## Conclusion

We proposed and described a new genus and species of Caballerotrematidae infecting *A. gigas sensu lato* from the Amazon River, Colombia. We accepted two caballerotrematid species infecting arapaimas (*C. brasiliense* and *A. sandrae*), synonymized *C. piscicola* with *C. brasiliense*, reassigned *C. annulatum* to *Alobophora*, and provided a dichotomous key to Caballerotrematidae. The biodiversity of trematodes infecting bony tongues and their relatives (Osteoglossiformes) is undersampled. The parasites of these fishes have the potential to aid in testing hypotheses regarding host biogeography and natural history.
